# Prevalence and associations of asteroid hyalosis: the Beijing Eye Study

**DOI:** 10.1186/s12886-022-02586-6

**Published:** 2022-09-06

**Authors:** Chuan Zhang, Lei Shao, Li Dong, Wen Da Zhou, Rui Heng Zhang, Wen Bin Wei

**Affiliations:** Key Laboratory of Intraocular Tumor Diagnosis and Treatment, Beijing Ophthalmology & Visual Sciences Key Lab, Medical Artificial Intelligence Research and Verification Key Laboratory of the Ministry of Industry and Information Technology, Beijing Tongren Eye Center, Beijing Tongren Hospital, Capital Medical University, Beijing, China

**Keywords:** Asteroid hyalosis, Prevalence, Associations, The Beijing Eye Study

## Abstract

**Background:**

To determine the prevalence and associations of asteroid hyalosis (AH) in a Chinese population-based cohort.

**Methods:**

The retrospective, cross-sectional, population-based Beijing Eye Study 2011 included 3468 individuals with a mean age of 64.6 ± 9.8 years (range: 50–93 years). Participants underwent detailed ophthalmic examinations including fundus photographs for diagnosis of AH. Data on systemic and ocular factors were collected for all participants according to the standardized protocol. Multiple linear regression and multivariate Logistic regression analysis were performed.

**Results:**

Fundus photographs were gradable in 3419 subjects. AH was detected in 63 (0.9%, 95% CI: 0.7%, 1.1%) eyes of 53 (1.6%, 95% CI: 1.1%, 2.0%) subjects. AH was bilateral in 18.9%. Mean age of all subjects with AH was 69.2 ± 9.5 years (median, 71.0 years; range, 51–91 years), mean spherical equivalent was 0.63 ± 1.53D (median, 0.75 D; range, -4.12 to 4.00D). In multivariate analysis, prevalence of AH was associated with elder age (*P* = 0.014, OR 1.057), thicker lens (*P* = 0.032, OR 3.887), higher spherical equivalent (*P* = 0.017, OR 1.396).

**Conclusions:**

In adult Chinese in Beijing, the prevalence of AH was 0.9% for eyes or 1.6% for subjects. AH was associated with elder age, thicker lens, and higher spherical equivalent. It was not associated with diabetes or other systemic indicators.

## Introduction

Asteroid hyalosis (AH) is a condition in which small yellow-white, spherical particles known as asteroid bodies (ABs) are distributed throughout the vitreous either randomly or in chains or sheets. ABs, which are composed of lipids complexed with calcium, phosphates, and oxygen [1], have a smooth spherical morphology and move with the displacement of the vitreous during head or eye movement. Compared with vitreous opacity, AH usually has less effect on vision [2] and does not require intervention. Learning the predisposing conditions of AH may provide clues for determining the formation mechanism of ABs.

Earlier reports have associated AH with systemic conditions, including diabetes [3, 4], hyperlipidemia [5, 6], hypercholesterolemia [6], and hypertension [3]. There has been no population-based investigation on the occurrence and associations of AH with other ocular and general parameters in the Chinese population. We therefore conducted the present study to describe the prevalence of AH in a representative older Chinese population and to investigate systemic and ocular associations. We also explored the relationship with systemic indicators that have not yet been reported, such as waist circumference, blood pressure, smoking and snoring, which have been proved to be related to hypertension, diabetes, and other systemic vascular changes.

## Methods

### Ethics statement

The Medical Ethics Committee of the Beijing Tongren Hospital approved the study protocol and all participants gave informed written consent, according to the Declaration of Helsinki.

The Beijing Eye Study 2011 is a population-based cross-sectional study in Beijing, China. The only eligibility criterion for inclusion in the study was an age of over 50 years. In 2011, it had a total population of 4403 individuals aged 50 years or older. Information on demographic variables, socioeconomic background, and known major systemic diseases were collected. Fasting blood samples were taken for measurement of blood lipids, serum creatinine, glucose and glycosylated hemoglobin HbA1c. Blood pressure was measured. Body height and weight and the circumference of the waist and hip were recorded. Systolic and diastolic blood pressures were the average of two measurements. Hypertension was defined as a systolic blood pressure of 140 mmHg or higher, a diastolic blood pressure of 90 mmHg or higher, or a history of hypertension with the use of antihypertension drugs [7]. The diagnostic criteria of diabetes were that the glycosylated hemoglobin value was over 6.5%, the fasting blood glucose level was over 7.0 mmol/l, or a history of diabetes treated with insulin, oral hypoglycemic agents, or diet, or newly diagnosed during participation in the study [8]. The diagnostic criteria of hyperlipidemia refered to relevant international guidelines [9]. Current or exsmokers are those who had smoked at least 100 cigarettes in their lifetime.

All subjects underwent comprehensive ophthalmological examination. Visual acuity (VA), best corrected VA (Auto Refractometer AR-610, Nidek Co., Ltd, Tokyo, Japan), intraocular pressure (IOP, Goldmann tonometer) were recorded before pupil dilatation. The slit lamp examination, indirect ophthalmoscope examination, anterior segment (Lenstar 900® Optical Biometer, Haag-Streit, 3098 Koeniz, Switzerland), fundus examination (spectral-domain OCT, Optovue Inc. Fremont, CA, U.S.A.; Spectralis, Heidelberg Engineering, Heidelberg, Germany) were performed. The patient underwent macular scanning by spectral-domain OCT. The examination includes 100 average scans, with the fovea as the center and the rectangular angle of 5° -30°, a total of 7 scans. After obtaining the scanned image, the data including central macular thickness were measured with Heidelberg eye Explorer software (version 5.3.3.0; Heidelberg engineering company, Germany). The spherical equivalent (SE) was calculated according to the format: SE = spherical degrees + (cylindrical degrees / 2). Posterior vitreous detachment (PVD) was defined as the separation of the posterior vitreous cortex from the inner surface of the retina observed in spectral-domain OCT. The study has been described in detail [10].

**Fig. 1:**
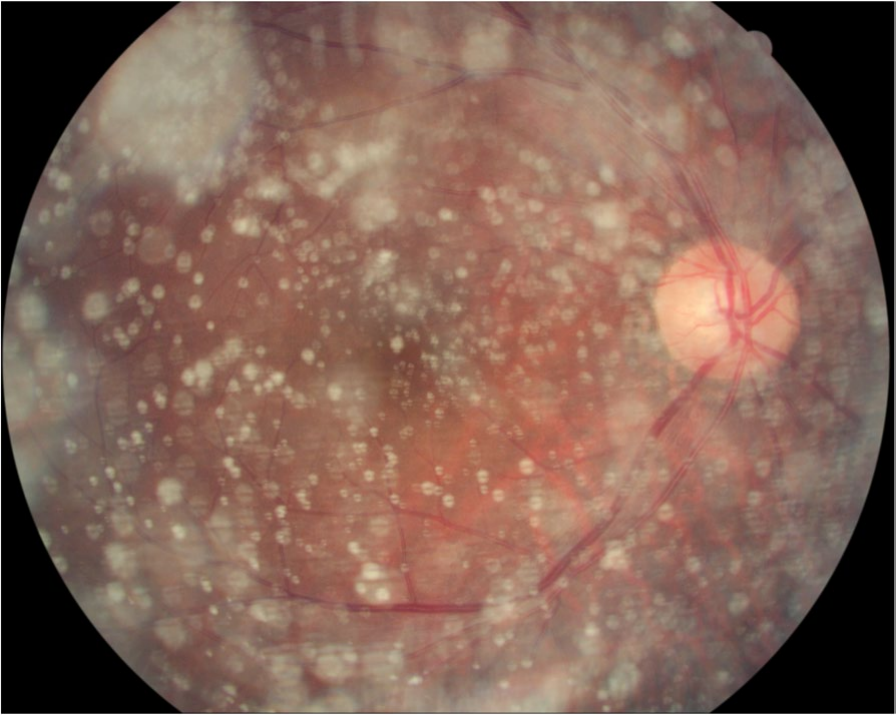
A fundus image of asteroid hyalosis

The assessment included stereoscopic 30° and 45°retinal photographs, using a fundus camera (Type CR6-45NM, Canon Inc. U.S.A.) of multiple fields in both eyes. Signs of AH were recorded either from the presence of typical ABs seen at the slit-lamp or on retinal examination of the indirect ophthalmoscope or from the stereo fundus images (Fig. 1). The images were taken by one technician (CXC) and the images were assessed by experienced and trained ophthalmologists (CZ, LS). In case of doubt, the fundus images were reassessed by a panel including several ophthalmologists (LS, WBW).

The statistical analysis was performed using a commercially available statistical software package (SPSS for Windows, version 25.0, SPSS, Chicago, IL, USA). The prevalence of AH was calculated. And we performed both univariate and multivariate logistic regression analyses to assess associations with AH. 95% Confidence intervals (CI) and odds ratio (OR) was presented. All *P*-values were 2-sided and were considered statistically significant when the values were less than 0.05.

## Results

**Fig. 2:**
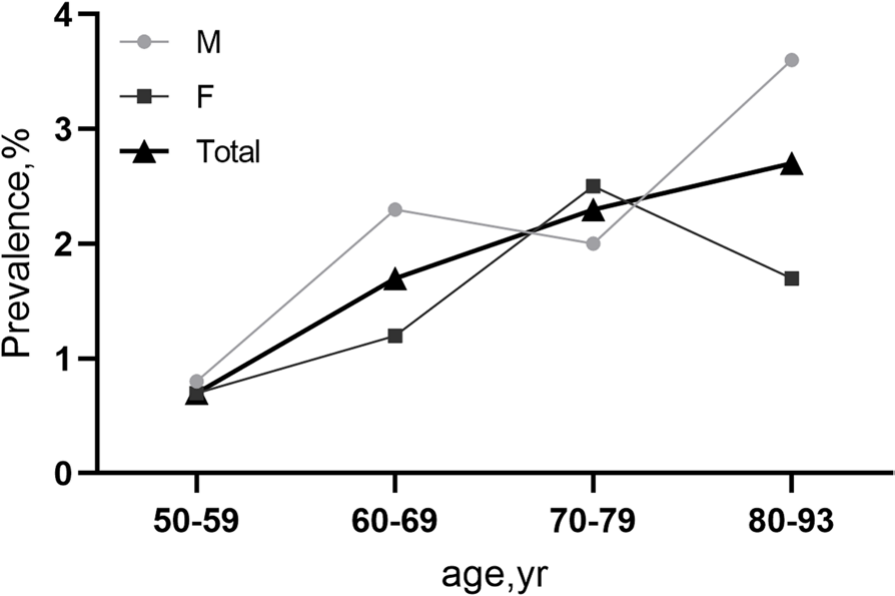
Prevalence of asteroid hyalosis by age and sex groups in Beijing Eye Study 2011.(M, male; F, female; yr, year.)

In total, 3468 individuals (1963 (56.6%) women) participated in the eye examination, corresponding to an overall response rate of 78.8%. Of 3468 participants in the Beijing Eye Study, 3419 had gradable fundus images were included in the study. 49 subjects were excluded because of their previous history of eye trauma, vitreoretinal surgery or inability to obtain clear fundus images. The mean age of the included subjects was 64.6 ± 9.8 years (median, 64 years; range, 50—93 years), and the mean SE was -0.18 ± 2.10 D (range, -22.0 to + 15.50 D). AH was detected in 63 (0.9%, 95% CI: 0.7%, 1.1%) eyes of 53 (1.6%, 95% CI: 1.1%, 2.0%) subjects, including 22 right, 21 left, and 10 for both eyes. Prevalence increased significantly with age from 0.7% in the 50–59 year age group to 2.7% in the 80–93 year age group (Fig. 2). AH was bilateral in 18.9% (10/53). The mean age of all subjects with AH was 69.2 ± 9.5 years (median, 71.0 years; range, 51–91 years), mean SE was 0.63 ± 1.53D (median, 0.75 D; range, -4.12 to 4.00D).

In univariate analysis, the prevalence was significantly associated with the systemic parameters of elder age (*P* = 0.001), and with the ocular parameters of higher SE (*P* = 0.034), thicker lens (*P* < 0.001), larger pupil diameter (*P* = 0.043). It was not significantly associated with the systemic parameters of gender (*P* = 0.267), body height (*P* = 0.566), weight (*P* = 0.380), the circumference of the waist (*P* = 0.963), the circumference of the hip (*P* = 0.696), diastolic blood pressure (*P* = 0.342), systolic blood pressure (*P* = 0.780), serum concentrations of low-density lipoproteins(*P* = 0.832), high-density lipoproteins (*P* = 0.407), cholesterol (*P* = 0.567), creatinin (*P* = 0.576), triglycerides (*P* = 0.899), glucose (*P* = 0.129), glycosylated hemoglobin (*P* = 0.245), diabetes (*P* = 0.829), hypertension (*P* = 0.390), hyperlipidemia (*P* = 0.532), self-reported diagnosis of cerebral infarction or haemorrhage (*P* = 0.683), and coronary heart disease (*P* = 0.264), frequency of reported snoring (*P* = 0.778), smoking (*P* = 0.357), drinking (*P* = 0.729). It was not significantly associated with demographic and socioeconomic variables of cognitive level (*P* = 0.882), and with the ocular parameters of anterior chamber depth (*P* = 0.648), central corneal thickness (*P* = 0.394), corneal diameter (*P* = 0.097), best corrected visual acuity (*P* = 0.356), posterior vitreous detachment (PVD) (*P* = 0.493), macular thickness (*P* = 0.207) (Table 1).

**Table 1:** Factors Associated with asteroid hyalosis in the Beijing Eye Study (Univariate analysis)

	AH group	Without AH group	*P*-value	t/χ^2^
Systemic Factors	*n* = 53	*n* = 3366		
Age (years)	69.16 ± 9.53	64.57 ± 9.81	0.001*	-3.389
Gender			0.267	1.255
Male	27 (1.8%)	1456 (98.2%)		
Female	26 (1.3%)	1910 (98.7%)		
Body height (cm)	161.25 ± 8.62	161.90 ± 8.17	0.566	0.574
Body weight (kg)	65.48 ± 11.51	66.90 ± 11.70	0.380	0.879
Circumference of the waist (cm)	88.70 ± 9.67	88.76 ± 10.36	0.963	-0.046
Circumference of the hip (cm)	99.23 ± 7.04	99.63 ± 7.48	0.696	-0.390
Cognitive level			0.882	0.043
High school and higher	16 (1.5%)	1036 (98.5%)		
Not reaching high school	35 (1.6%)	2127 (98.4%)		
Diastolic blood pressure (mmHg)	68.42 ± 12.83	70.06 ± 12.53	0.342	0.951
Systolic blood pressure (mmHg)	131.13 ± 24.32	130.33 ± 20.58	0.780	-0.280
Low-density lipoproteins (mmol/L)	3.33 ± 0.84	3.36 ± 0.92	0.832	0.212
High-density lipoproteins (mmol/L)	1.29 ± 0.32	1.48 ± 1.41	0.407	0.830
Cholesterol (mmol/L)	4.93 ± 0.90	5.04 ± 1.18	0.567	0.573
Creatinine (μmol/L)	70.90 ± 16.77	67.96 ± 27.73	0.576	-0.559
Triglycerides (mmol/L)	1.68 ± 0.86	1.73 ± 2.48	0.899	0.127
Glucose (mmol/L)	6.14 ± 2.10	5.61 ± 1.59	0.129	-1.553
Glycosylated hemoglobin (%)	4.55 ± 1.21	4.35 ± 1.03	0.245	1.163
Diabetes			0.829	0.078
No	40 (1.6%)	2412 (98.4%)		
Yes	7 (1.8%)	376 (98.2%)		
Hypertension			0.390	0.816
No	21 (1.4%)	1472 (98.6%)		
Yes	28 (1.8%)	1510 (98.2%)		
Known coronary disease			0.264	1.249
No	33 (1.4%)	2398 (98.6%)		
Yes	11 (2.0%)	541 (98.0%)		
Known hyperlipidemia			0.532	0.604
No	34 (1.5%)	2266 (98.5%)		
Yes	17 (1.9%)	898 (98.1%)		
Cerebral infarction or haemorrhage			0.683	0.167
No	45 (1.5%)	2282 (98.5%)		
Yes	3 (1.3%)	226 (98.7%)		
Smoking			0.357	1.104
No	39 (1.7%)	2204 (98.3%)		
Current or exsmoker	12 (1.2%)	960 (98.8%)		
Alcohol consumption			0.729	0.177
40 g per week or less	39 (1.5%)	2497 (98.5%)		
Over 40 g per week	12 (1.8%)	668 (98.2%)		
Snoring			0.778	0.107
No or mildly	21 (1.6%)	1327 (98.4%)		
Snore moderately or severely	30 (1.7%)	1726 (98.3%)		
Ocular Factors	*n* = 63	*n* = 6775		
Spherical equivalent (D)	0.39 ± 1.54	-0.19 ± 2.10	0.034*	2.115
Lens thickness (mm)	4.85 ± 0.44	4.56 ± 0.33	0.000*	4.467
Pupil diameter (mm)	3.77 ± 0.60	4.08 ± 0.80	0.043*	-2.023
Anterior chamber depth (mm)	2.45 ± 0.72	2.49 ± 0.49	0.648	-0.456
Central corneal thickness (μm)	527.24 ± 29.71	532.41 ± 32.56	0.394	-0.852
Corneal diameter (mm)	12.27 ± 0.66	11.94 ± 1.00	0.097	1.659
Best corrected visual acuity (LogMAR)	0.87 ± 0.34	0.92 ± 0.24	0.356	-0.936
Posterior vitreous detachment			0.493	1.587
No	20 (0.8%)	2537 (99.2%)		
Partial	39 (1.1%)	3581 (98.9%)		
Yes	2 (0.7%)	277 (99.3%)		
Macular thickness (μm)	228.13 ± 32.21	221.80 ± 39.09	0.207	1.261

*Cm* Centimeter, *mm* Millimeter, *μm* Micrometer, *kg* Kilogram, *D* Diopter

We then performed multivariate logistic regression analysis on variables with statistical significance and/ or clinical significance in univariate analysis. In a multivariate logistic regression analysis (forward stepwise method), variables with *P* < 0.1 in univariate analysis (age, SE, lens thickness, pupil diameter (*P* < 0.05), corneal diameter (*P* = 0.097)) and risk factors reported in previous studies (glucose (*P* = 0.129), cholesterol (*P* = 0.567), triglycerides (*P* = 0.899)) were included. The prevalence of AH was no longer significantly associated with pupil diameter (*P* = 0.283). The associations between the presence of AH and elder age (*P* = 0.014, OR 1.057), thicker lens (*P* = 0.032, OR 3.887), and higher SE (*P* = 0.017, OR 1.396), were statistically significant. The prevalence of AH was not significantly associated with the systemic parameters of glucose, cholesterol, and triglycerides (Table 2).

**Table 2:** Factors associated with asteroid hyalosis using multivariate logistic regression models in the Beijing Eye Study

Factor	*P*-value	OR	95% confidence interval	
Age	0.014	1.057	1.011,	1.105
Lens thickness	0.032	3.887	1.121,	13.476
Spherical equivalent	0.017	1.396	1.062,	1.834
Glucose (mmol/L)	0.757			
Cholesterol (mmol/L)	0.499			
Triglycerides (mmol/L)	0.817			
Pupil diameter	0.283			
Central corneal thickness	0.622			

## Discussion

In our population-based study on adult Chinese in Beijing, the prevalence of AH was 0.9% per eye or 1.6% per subject. AH was associated with elder age (*P* = 0.014, OR 1.057), thicker lens (*P* = 0.032, OR 3.887), and higher SE (*P* = 0.017, OR 1.396). AH was not associated with the systemic parameters of blood pressure, glucose, diabetes, cholesterol, and triglycerides.

Previous studies of the prevalence of AH were in autopsy series and clinical populations [11]. However, the best source for prevalence estimates in a general population is population-based studies. To our knowledge, this is the first large population-based study reporting on the prevalence of AH in Asian populations. Our prevalence (1.6%, 95% CI: 1.1%, 2.0%) results are consistent with previous studies of AH [12]. In the Beaver Dam Eye Study [13] of 4,952 patients, AH was present in 1.1% (95% CI: 0.9%, 1.5%) of the total population. The Australian Blue Mountains Eye Study [14] of 3,654 patients confirmed a similar 1.0% (95% CI:0.7%, 1.3%) prevalence of AH. In the autopsy series of 10,801 eyes studied at UCLA [15], AH had a prevalence of 1.96%.

Our data show that AH was associated with elder age (*P* = 0.014). Prevalence increased significantly with age from 0.7% in the 50–59 year age group to 2.7% in the 80–93 year age group. Several clinical-based studies [13, 15, 16] concluded that the occurrence of AH was significantly associated with elder age. In the Beaver Dam Eye Study [13], the prevalence of AH increased from 0% of persons aged less than 55 years to 2.1% of persons aged 75 years or older. In the Australian Blue Mountains Eye Study [14], the prevalence of AH also increased with age from 0.2% in subjects 43–54 years, to 2.9% in subjects 75–86 years. These findings confirm our results. Komatsu and coworkers [17] used samples of AH obtained in vitreous surgery and observed with light and electron microscopes and processed by the focused ion-beam method. They thus concluded that AH was produced not only by changes in ionic tension in the vitreous fluid but also by changes in the vitreous matrix in the aging process and diseases.

Our data also show that AH was associated with a higher SE (*P* = 0.017). Bergren [3] and coworkers performed a cross-sectional study of 12,205 patients and reported patients with AH were more hyperopic than control subjects. Studies have shown that patient with AH presented with stronger vitreoretinal adhesion [18, 19]. One explanation is that the complete posterior vitreoretinal interface may be important for the formation of asteroid zona pellucida. Also, the presence of ABs may prevent the process of vitreous collapse or contraction [20]. Therefore, individuals who maintain posterior vitreous attachment are more likely to develop AH. This may explain why AH is associated with a hyperopic SE because PVD is more common in myopia and occurs at an earlier age [21]. However, we did not find an association between AH and PVD (*P* = 0.417) in our study. Another possibility is that the presence of AH may arrest the process of vitreous collapse or contraction and has a protective effect on vitreous liquefaction [22], which prevents PVD.

In our population-based study, AH also correlated to the thicker lens (*P* = 0.032). As there is no relevant report so far, the associations between AH and lens thickness have remained unclear. One of the reasons may be that the lens thickness was associated with higher age and hyperopic SE [23]. Previous studies [24] have proved that the age-related increase in lens thickness was due to the continuous production of new lens fibers in the equatorial region of the lens. Besides, the higher refractive power necessary in hyperopic eyes lead to greater lens thickness [24]. From a geometric point of view, the thicker lens partially protrudes forward into the anterior chamber and partially bulged backward into the vitreous cavity, which may affect the dynamics of aqueous humor circulation [25] and thus cause changes in the composition of the vitreous extracellular matrix.

In our study, AH was not associated with diabetes. The prevalence of diabetes was higher in AH group (1.8% vs. 1.6%), the glucose level was higher in AH group (6.14 ± 2.10 vs. 5.61 ± 1.59, mmol/l), and the glycosylated hemoglobin level was higher in AH group (4.55 ± 1.21 vs. 4.35 ± 1.03, %), but there was no significant difference (*p* = 0.829, 0.129, 0.245, respectively). In fact, except for a few retrospective small sample studies [20, 26] that reported the correlation between AH and diabetes, most of the large sample studies (The Blue Mountains Eye Study [14], *n* = 3654; The Yonsei Eye Study [2], n = 13,016; The Beaver Dam Eye Study [13], *n* = 4926) reported that there was no statistical correlation between AH and diabetes. Fawzi et al. reviewed 10,801 patients in the University of California at Los Angeles (UCLA) autopsy eye database and reported that there was no correlation between diabetes and AH [15]. They found only specific age subgroups (51–60 years, *P* = 0.006; 41–60 years, *P* = 0.004) that showed a statistically significant association between AH and diabetes. However, there was no correlation among the age groups younger than 40 years, 81–90 years, and 91 years and over (*P* = 0.50, 0.53, and 0.73, respectively.). Considering the irreversibility of the course of diabetes, we have reason to doubt the accuracy of this result. Elbaz et al. reported that the association between diabetes and AH was substantially attenuated from a univariate OR of 3.88 to an OR of 1.99 after adjustment for sex and age [4]. The relationship between AH and diabetes may be explained by the increase of basal membrane permeability in patients with diabetes, which are likely to be the source of phospholipids and calcium required for asteroid formation [27]. But the current data cannot be conclusive, further physiology research is needed.

In our study, AH was not associated with other systemic parameters of body height, weight, the circumference of the waist, the circumference of the hip, diastolic blood pressure, systolic blood pressure, serum concentrations of low-density lipoproteins, high-density lipoproteins, cholesterol, creatinin, triglycerides, glucose, glycosylated hemoglobin, diabetes, hypertension, hyperlipidemia, self-reported diagnosis of cerebral infarction or haemorrhage, and coronary heart disease, frequency of reported snoring, smoking. AH has purportedly been associated with several systemic diseases, including diabetes [5], hypercholesterolemia [5], hypertension [3], hypercalcemia [26], and gout [16]. Many of these studies have been case series, case–control studies, or performed in clinic populations and may reveal a selection bias for diseases that are more prevalent in patients presenting for ophthalmic and vitreoretinal evaluations. Thus, these findings cannot readily be extrapolated to the general population. Besides, the prevalence of bilateral AH was 18.9% in our study. The fact that AH mainly occurs unilaterally [3, 13–15] does not support an association with systemic parameters.

Attention should be paid to AH, since it may prevent PVD and have a protective effect [22] on vitreomacular traction. Besides, AH generally only has a minor impact on vision and thus may be a useful model for better understanding the interaction between incident light and intravitreal structures. Further studies on AH may help in understanding the pathogenesis of ABs by showing the correlations between AH and other ocular and general parameters.

This is the first population-based investigation searching for the prevalence and associations between ocular and systemic parameters and AH in the Chinese population. Despite the advantages of this population-based study, potential limitations of our study should be mentioned. First, a major concern regarding any prevalence study is non-participation. The Beijing Eye Study 2011 had a reasonable response rate of 78.8%, although differences between participants and non-participants can lead to a selection bias. Second, the presence of AH was determined either from the presence of typical ABs seen at the slit-lamp or on retinal examination or from the stereo fundus images of a limited number of fields. ABs may present only in peripheral areas and not be detected. Thus, we are probably underestimating the prevalence of AH. Another limitation is the cross-sectional design of the study. This prevents us from knowing the antecedent-consequent relationship between the risk factors and the endpoint.

In conclusion, in adult Chinese in Beijing, the prevalence of AH was 0.9% for eyes or 1.6% for subjects. AH was associated with elder age, thicker lens, and higher SE.

## Data Availability

The datasets used and/or analysed during the current study are available from the corresponding author on reasonable request.
